# Negative-Pressure Pulmonary Hemorrhage: A Case Report and Review of Differential Diagnosis

**DOI:** 10.7759/cureus.107032

**Published:** 2026-04-14

**Authors:** Anna Theodorou-Kanakari, Styliani Karathanasi, Ioannis Kougioumtzis, Agamemnon Katsioulas, Konstantinos Ganas

**Affiliations:** 1 Anaesthesiology, General Hospital of Nikaia - Piraeus, Nikaia, GRC; 2 Sports Medicine and Orthopaedic Traumatology, General Hospital of Nikaia - Piraeus, Nikaia, GRC; 3 Intensive Care Unit, General Hospital of Nikaia - Piraeus, Nikaia, GRC

**Keywords:** case report, differential diagnosis, emergence from anesthesia, negative-pressure pulmonary edema, negative-pressure pulmonary hemorrhage

## Abstract

Negative-pressure pulmonary hemorrhage (NPPH) represents an uncommon complication of acute upper airway obstruction. During emergence from anesthesia, strenuous inspiratory efforts can generate profound negative intrathoracic pressure. This mechanism not only drives alveolar fluid extravasation, leading to negative-pressure pulmonary edema, but can also precipitate capillary stress failure and subsequent alveolar hemorrhage. We present the case of a healthy young patient who developed NPPH following partial strangulation without obvious tube occlusion or laryngospasm. This report discusses the diagnostic challenges, differential considerations, and the supportive management that led to a rapid resolution of symptoms.

## Introduction

Negative-pressure pulmonary hemorrhage (NPPH) is a rare but potentially life-threatening complication of general anesthesia, with an estimated incidence ranging from 0.01% to 0.05% [1]. It typically occurs following an episode of upper airway obstruction and is considered a severe manifestation within the spectrum of negative-pressure pulmonary syndromes, which also includes negative-pressure pulmonary edema (NPPE) [1]. While NPPE is more commonly encountered and characterized by fluid transudation into the alveolar space, NPPH involves disruption of the alveolar-capillary membrane with subsequent bleeding into the alveoli [2]. The underlying pathophysiology is related to the generation of markedly negative intrathoracic pressures, leading to increased pulmonary capillary transmural pressure and capillary stress failure [2]. Clinically, NPPH presents with acute respiratory deterioration, hypoxemia, and hemoptysis, typically in the immediate postoperative period, although delayed presentations have also been described [2]. We present a case of NPPH in a previously healthy young patient occurring immediately following emergence from anesthesia, highlighting the diagnostic challenges and the importance of early recognition and supportive management. Written informed consent was obtained from the patient for publication of this case and accompanying images.

## Case presentation

An 18-year-old male, smoker, with a body mass index of 22.5 kg/m² and no significant past medical history or known allergies, was admitted for elective anterior cruciate ligament (ACL) reconstruction following a motorcycle accident knee injury. The patient reported a recent upper respiratory tract infection 10 days before surgery, treated conservatively.

Anesthesia was induced with propofol 200 mg, fentanyl 150 µg, and rocuronium 60 mg, while desflurane and remifentanil were used for anesthesia maintenance. Adjunct medications administered intraoperatively included dexamethasone 8 mg, ondansetron 4 mg, paracetamol 1 g, ibuprofen 400 mg, and pethidine 30mg. The surgical procedure lasted approximately 2.5 hours. A pneumatic tourniquet was applied to the operative leg one hour after intubation and was released immediately before emergence from anesthesia. Neuromuscular blockade was reversed with sugammadex 200 mg. During emergence, the patient became agitated, coughing against the endotracheal tube but without obvious tube biting. This behavior caused forceful inspiratory efforts and resulted in a transient partial upper airway obstruction. To facilitate smoother emergence, propofol 40 mg was administered. Approximately two minutes later, the patient was extubated uneventfully, in a cooperative state. Upon removal of the endotracheal tube, pink-tinged speckled secretions were noted within its lumen, prompting concern among the anesthesiology team for an acute pulmonary process.

**Figure 1:**
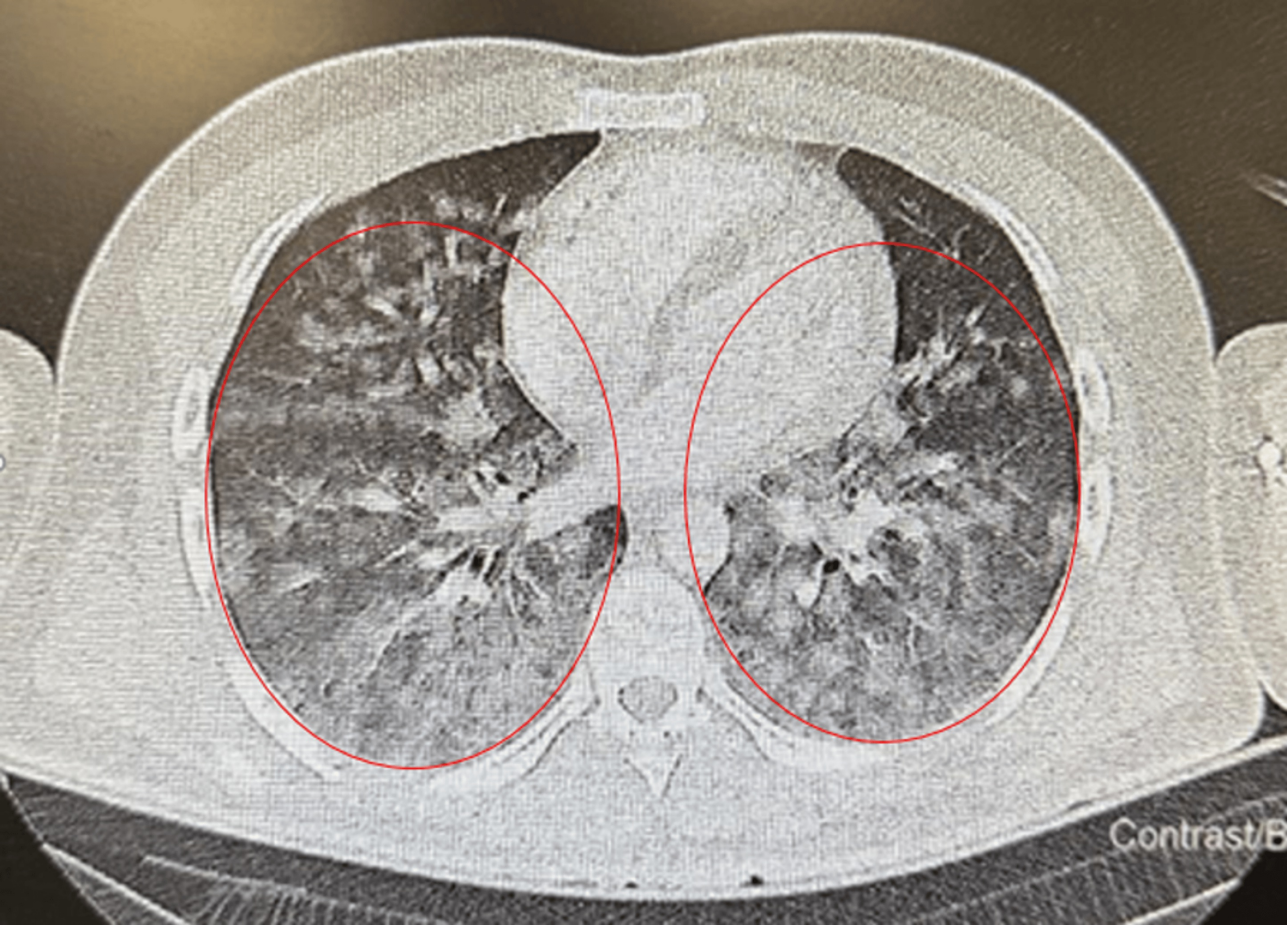
CTPA revealing bilateral diffuse ground-glass opacities and central alveolar infiltrates, with no evidence of pulmonary embolism. Axial computed tomography pulmonary angiography (CTPA) demonstrating bilateral diffuse ground-glass opacities and central alveolar infiltrates (circled), with no evidence of pulmonary embolism.

In the post-anesthesia care unit, a 40% Venturi mask was applied to the patient, who initially communicated well and did not report dyspnea. Shortly thereafter, a rapid oxygen desaturation was observed (SpO₂ of 80%). Oxygen flow was subsequently increased to 100%, which improved SpO₂ to 98%. Lung auscultation revealed normal breath sounds bilaterally, without crackles, wheeze, or signs of bronchospasm. After approximately 20 minutes, the patient experienced clinical deterioration, developing acute dyspnea, tachypnea with a respiratory rate of 30 breaths per minute, and sinus tachycardia at 130 beats per minute. This was accompanied by a single episode of hemoptysis of small volume. Progressive clinical decline prompted an arterial blood gas analysis, which revealed a PaO₂ of 160 mmHg on FiO₂ 100%, indicating severe impairment of oxygenation (P/F ratio of 160). Given the acute onset and severity, the patient was transferred for an emergent computed tomography pulmonary angiography (CTPA) to exclude pulmonary embolism, and subsequently admitted to the intensive care unit (ICU). CTPA identified no emboli; however, it demonstrated diffuse bilateral ground-glass opacities and radiologic features consistent with diffuse alveolar injury or pulmonary edema (Figure 1).

**Figure 2:**
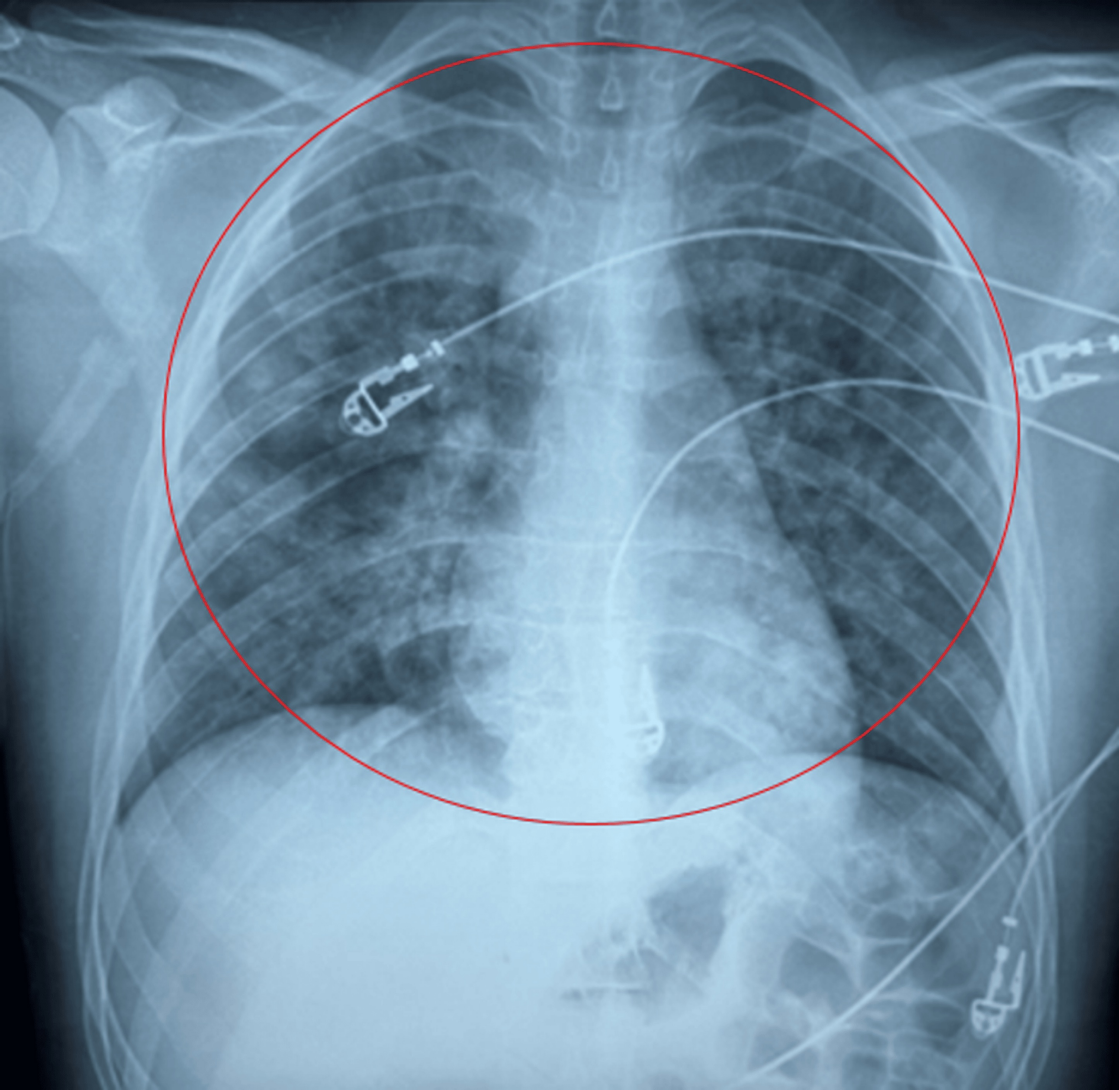
Chest radiograph on ICU admission showing diffuse bilateral pulmonary infiltrates. Chest radiograph on intensive care unit (ICU) admission demonstrating diffuse bilateral pulmonary infiltrates (circled), with predominant involvement of the perihilar and lower lung fields, consistent with a diffuse alveolar process.

Upon ICU admission, a nasopharyngeal multiplex respiratory viral panel (FilmArray) and SARS-CoV-2 testing were negative. The patient was hemodynamically stable, afebrile, and fully communicative, while blood investigations were within normal limits. Serial hemoglobin measurements demonstrated minimal variation (from 14.3 g/dL preoperatively to 13.9 g/dL in the ICU), further supporting the absence of ongoing or clinically significant bleeding. Oxygen saturation was 93%, heart rate was 153 beats per minute, and blood pressure was 130/90 mmHg. Lung auscultation revealed bilateral crackles. A follow-up chest X-ray was obtained (Figure 2).

**Figure 3:**
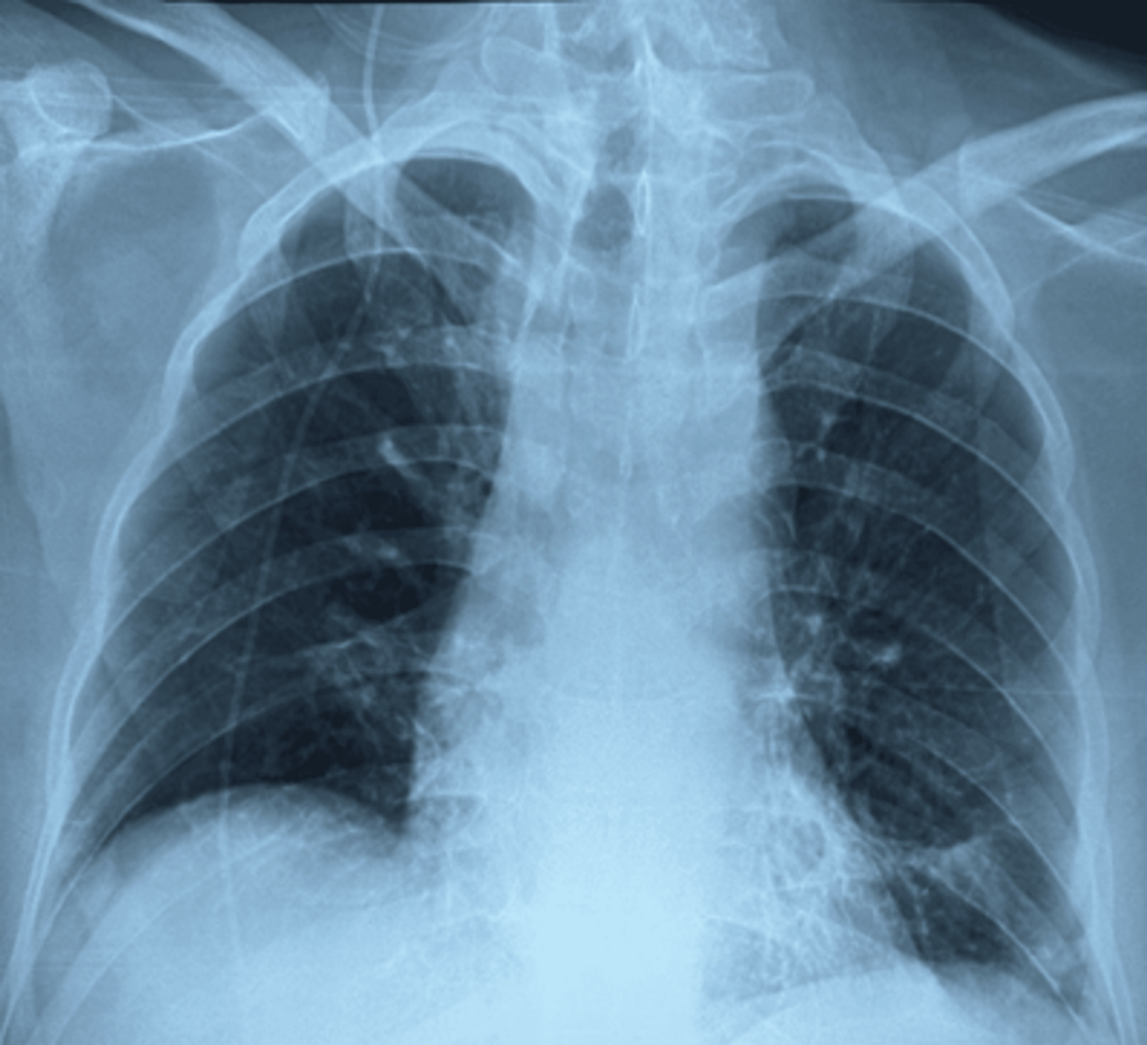
Chest radiograph on ICU discharge demonstrating resolution of bilateral pulmonary infiltrates. Chest radiograph on intensive care unit (ICU) discharge demonstrating significant resolution of the previously noted bilateral pulmonary infiltrates. Marked radiographic improvement compared to the admission study is consistent with the expected course of negative-pressure pulmonary hemorrhage.

Management consisted of conservative, supportive measures with high-flow supplemental oxygen, systemic corticosteroids, empiric antibiotic therapy, ipratropium nebulizer, tranexamic acid 1 g, and monitoring. No mechanical ventilation was required. No other episode of hemoptysis occurred. The patient demonstrated rapid clinical improvement within 24 hours, with complete resolution of hypoxemia, which was paralleled by significant radiologic improvement on follow-up imaging (Figure 3). He was discharged from the ICU after 48 hours and went home two days later in good condition, without any residual respiratory symptoms.

## Discussion

This case illustrates a classic presentation of NPPE, occurring with such severity that it progressed to frank NPPH. NPPE is caused by the generation of markedly negative intrathoracic pressures during forceful inspiration against a closed or partially obstructed airway. These excessive negative pressures increase venous return and pulmonary capillary hydrostatic pressure while simultaneously lowering alveolar pressure, creating a large transcapillary gradient that disrupts the alveolar-capillary membrane and results in alveolar flooding and pulmonary edema [2]. Young, athletic patients, such as this patient, are particularly susceptible due to strong inspiratory musculature [3]. They can generate very high levels of negative inspiratory pressure with a maximum of −140 cmH₂O [4].

Common triggers for NPPE include airway lesions, endotracheal tube biting, foreign body, strangulation, epiglottitis, and, most notably, post-extubation laryngospasm [5]. Risk factors such as obesity, obstructive sleep apnea, short neck, and ENT procedures further predispose patients to obstruction [6]. In our case, the patient’s history of smoking and a recent upper respiratory tract infection significantly increased airway irritability, probably contributing to the development of NPPE and NPPH. Hence, agitation at emergence can precipitate a “functional” airway obstruction.

Although NPPE is typically associated with post-extubation laryngospasm and endotracheal tube biting, our case demonstrates that its development may occur despite a visibly patent endotracheal tube in situ. Secretions within the endotracheal tube or kinking of the tube in the oropharynx can result in partial obstruction [5]. In our case, a plausible explanation, grounded in physical laws, is that increased inspiratory effort against such partial obstruction generated markedly negative intrathoracic pressure. According to Poiseuille’s law, the fixed internal diameter of the endotracheal tube (e.g., size 7.5) inherently limits flow capacity, so even minor reductions in effective radius can markedly increase airflow resistance and promote NPPE development. Furthermore, when a patient’s peak inspiratory flow demand, potentially exceeding 100-120 L/minute during emergence agitation, surpasses the laminar flow limits of the tube, the flow becomes highly turbulent. In this regime, the pressure-flow relationship is more accurately described by the Darcy-Weisbach equation: ΔP = f ∙ L/D ∙ ρυ²/2.

This formula demonstrates that the pressure drop (ΔP) across the endotracheal tube increases with the square of the gas velocity (υ²), creating a significant “functional” obstruction [7]. The strenuous effort to inhale through this high-resistance conduit acts as a Müller maneuver, generating the critical negative intrapleural pressures that lead to pulmonary capillary stress failure and ultimately to extravasation of red blood cells and manifestation of pulmonary hemorrhage (NPPH) [8]. The first NPPH case was reported in 1999 and was attributed to upper airway obstruction [9].

The sudden onset of postoperative respiratory distress accompanied by hemoptysis and bilateral radiographic opacities necessitates a broad differential diagnosis, including pulmonary embolism, anaphylaxis, fat embolism syndrome (FES), and acute respiratory distress syndrome (ARDS).

Pulmonary embolism remains a primary concern in patients presenting with acute postoperative dyspnea, hypoxemia, and tachycardia [10]. In this instance, however, pulmonary embolism was effectively ruled out by a negative CTPA. Similarly, an anaphylactic etiology, often triggered perioperatively by neuromuscular blocking agents, antibiotics, or sugammadex, was considered unlikely. The patient lacked the hallmark cutaneous manifestations, such as urticaria or angioedema, and remained hemodynamically stable without evidence of severe bronchospasm, typically associated with systemic hypersensitivity reactions [11].

Given the orthopedic context and the use of a pneumatic tourniquet, which has been linked to the systemic release of fat emboli [12], FES was initially considered. While the classic triad of respiratory distress, neurological dysfunction, and petechial rash is pathognomonic, it is only partially present in many cases [13]. Moreover, FES is a complication following long bone or pelvic fractures, and literature reveals no significant association between elective ACL reconstruction and FES. Although hypoxia is the most frequent finding (occurring in up to 96% of patients), respiratory symptoms in FES typically manifest 24-72 hours post-injury [13]. In our patient, the immediate onset of symptoms upon emergence and the absence of neurological or petechial signs argued strongly against FES.

Bilateral ground-glass opacities and hypoxemia observed in this patient are characteristic of ARDS. However, ARDS typically develops over hours to days following a systemic precipitant such as sepsis or aspiration. In contrast, this patient’s symptoms surfaced within minutes of extubation. Furthermore, the rapid clinical resolution within 24 hours under conservative management is uncharacteristic of ARDS, which generally requires prolonged ventilatory support [14].

The rapid onset following anesthesia emergence, the presence of hemoptysis, bilateral ground-glass opacities, and the prompt response to supportive therapy strongly support NPPH as the most likely diagnosis. Clinical hypoxemia plays a central role in raising suspicion for this condition. However, in the absence of a standardized diagnostic framework and given the inherently clinical nature of this diagnosis, NPPH is frequently considered a diagnosis of exclusion. In this case, alternative causes of acute respiratory deterioration, particularly pulmonary embolism, were excluded through negative CTPA findings. Bronchoscopy is often indicated to localize the bleeding source or exclude endobronchial pathology [15]. However, it was not performed in this case due to the isolated nature of hemoptysis and the patient’s rapid clinical improvement with conservative management. Preventive strategies, such as lidocaine administration before extubation to attenuate airway reflexes, thorough suctioning of secretions, and optimal timing of extubation, may reduce the incidence of NPPH [6].

## Conclusions

NPPH is a rare and potentially life-threatening postoperative complication that is often underrecognized and can be misdiagnosed as more common causes of acute respiratory deterioration. This case highlights the importance of considering NPPH in the differential diagnosis of sudden postoperative dyspnea and hemoptysis, even in the absence of clear airway obstruction, particularly in young, otherwise healthy patients. While the diagnosis in this case remains clinical and presumptive, early recognition and supportive management were associated with rapid clinical improvement. Further studies are required to better define the diagnostic approach and optimal management of this condition. Reporting such cases may contribute to increased awareness and improved perioperative patient safety.

## References

[1] Louis PJ, Fernandes R (2002). Negative pressure pulmonary edema. Oral Surg Oral Med Oral Pathol Oral Radiol Endod.

[2] Lemyze M, Mallat J (2014). Understanding negative pressure pulmonary edema. Intensive Care Med.

[3] Patton WC, Baker CL Jr (2000). Prevalence of negative-pressure pulmonary edema at an orthopaedic hospital. J South Orthop Assoc.

[4] Cook CD, Mead J (1960). Maximum and minimum airway pressures at various lung volumes in normal children and adults. Fed Proc.

[5] Shaikh N, Alali B, Amara UE, Nashrah UE, Ummunnisa F (2025). Negative-pressure pulmonary edema: a perioperative emergency. Cureus.

[6] Han IS, Han BM, Jung SY, Yoon JR, Chung EY (2018). Negative pressure pulmonary hemorrhage after laryngospasm during the postoperative period. Acute Crit Care.

[7] Yartsev A (2024). Yartsev A. Resistance of the respiratory system. https://derangedphysiology.com/main/cicm-primary-exam/respiratory-system/Chapter-035/resistance-respiratory-system.

[8] West JB (2000). Invited review: pulmonary capillary stress failure. J Appl Physiol (1985).

[9] Schwartz DR, Maroo A, Malhotra A, Kesselman H (1999). Negative pressure pulmonary hemorrhage. Chest.

[10] Desciak MC, Martin DE (2011). Perioperative pulmonary embolism: diagnosis and anesthetic management. J Clin Anesth.

[11] Dribin TE, Muraro A, Camargo CA Jr (2025). Anaphylaxis definition, overview, and clinical support tool: 2024 consensus report-a GA(2)LEN project. J Allergy Clin Immunol.

[12] Watanabe S, Terazawa K, Matoba K, Yamada N (2007). An autopsy case of intraoperative death due to pulmonary fat embolism--possibly caused by release of tourniquet after multiple muscle-release and tenotomy of the bilateral lower limbs. Forensic Sci Int.

[13] Rothberg DL, Makarewich CA (2019). Fat embolism and fat embolism syndrome. J Am Acad Orthop Surg.

[14] Bos LD, Ware LB (2022). Acute respiratory distress syndrome: causes, pathophysiology, and phenotypes. Lancet.

[15] Koch SM, Abramson DC, Ford M, Peterson D, Katz J (1996). Bronchoscopic findings in post-obstructive pulmonary oedema. Can J Anaesth.

